# Progress towards Stable and High-Performance Polyelectrolyte Multilayer Nanofiltration Membranes for Future Wastewater Treatment Applications

**DOI:** 10.3390/membranes13040368

**Published:** 2023-03-23

**Authors:** Áron Bóna, Ildikó Galambos, Nándor Nemestóthy

**Affiliations:** 1 Soós Ernő Research and Development Center, University of Pannonia, Vár u. 8., H-8800 Nagykanizsa, Hungary; 2 Research Institute on Bioengineering, Membrane Technology and Energetics, University of Pannonia, Egyetem u. 10., H-8200 Veszprém, Hungary

**Keywords:** nanofiltration, polyelectrolyte multilayer, membrane modification, layer-by-layer, membrane selectivity, membrane stability, wastewater treatment, micropollutant removal, fouling

## Abstract

The increasing demand for nanofiltration processes in drinking water treatment, industrial separation and wastewater treatment processes has highlighted several shortcomings of current state-of-the-art thin film composite (TFC NF) membranes, including limitations in chemical resistance, fouling resistance and selectivity. Polyelectrolyte multilayer (PEM) membranes provide a viable, industrially applicable alternative, providing significant improvements in these limitations. Laboratory experiments using artificial feedwaters have demonstrated selectivity an order of magnitude higher than polyamide NF, significantly higher fouling resistance and excellent chemical resistance (e.g., 200,000 ppmh chlorine resistance and stability over the 0–14 pH range). This review provides a brief overview of the various parameters that can be modified during the layer-by-layer procedure to determine and fine-tune the properties of the resulting NF membrane. The different parameters that can be adjusted during the layer-by-layer process are presented, which are used to optimize the properties of the resulting nanofiltration membrane. Substantial progress in PEM membrane development is presented, particularly selectivity improvements, of which the most promising route seems to be asymmetric PEM NF membranes, offering a breakthrough in active layer thickness and organic/salt selectivity: an average of 98% micropollutant rejection coupled with a NaCl rejection below 15%. Advantages for wastewater treatment are highlighted, including high selectivity, fouling resistance, chemical stability and a wide range of cleaning methods. Additionally, disadvantages of the current PEM NF membranes are also outlined; while these may impede their use in some industrial wastewater applications, they are largely not restrictive. The effect of realistic feeds (wastewaters and challenging surface waters) on PEM NF membrane performance is also presented: pilot studies conducted for up to 12 months show stable rejection values and no significant irreversible fouling. We close our review by identifying research areas where further studies are needed to facilitate the adoption of this notable technology.

## 1. Introduction

### 1.1. Nanofiltration

Nanofiltration (NF) is a pressure-based separation process where the apparent pore size is in the nanometer range (which motivated Eriksson to coin the term nanofiltration in 1984) [1]. It is between reverse osmosis (RO) and ultrafiltration (UF) in the filtration spectrum, tighter NF membranes being best described as a “loose reverse osmosis”, and their separation mechanism is best described by the solution diffusion model. On the other hand, size exclusion becomes more dominant for looser NF membranes which have molecular weight cutoff (MWCO) values close to 1 kDa (where the line is usually drawn between NF and UF), but charge-based rejection is still very important throughout the NF spectrum [2]. The complex rejection mechanism of NF membranes yields a great opportunity for separation applications, where selective rejection of certain compounds (e.g., softening, sulfate reduction, transition metal removal, etc.) is needed, combined with a high passage rate for others (e.g., NaCl) [3]. The higher specific water flux and the lower rejection of monovalent salts lowers the energy demand of nanofiltration compared to RO [2,3,4,5,6,7]. With current RO membranes achieving a high permeability [8], the main reason to choose NF over RO is the higher selectivity: in many cases, having a high passage of some solutes (e.g., monovalent salts) is an important feature [9].

NF offers great capability as a selective and green separation process for various industries. Drinking water regulations are getting stricter; therefore, tighter filtration processes than sand filtration or UF are preferred [10,11]. Compared to RO, the lower monovalent salt rejection and the higher water flux (in relation to this, the lower energy demand), combined with a reasonably high organic rejection, make NF an ideal process for drinking water treatment where high NOM concentrations pose a problem [12,13]. Climate change is causing an increase of dissolved organic matter in surface waters, especially in boreal regions, where these are important sources of drinking water [14,15].

**Figure 1:**
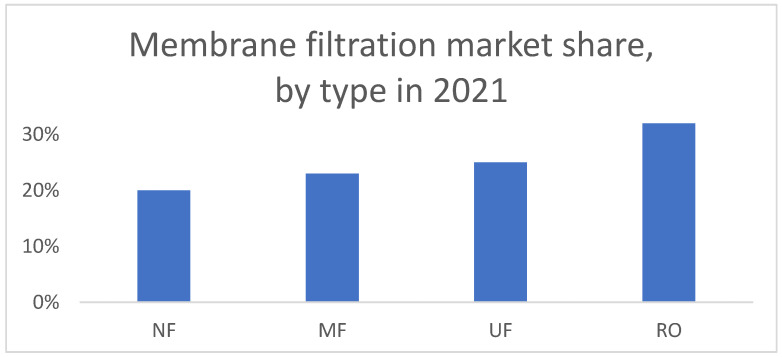
The market share of membrane types in 2021 [18].

There is also a growing need to clean the effluents of municipal wastewater treatment plants (WWTPs) from micropollutants (MP) and micro- and nanoplastics, which presents similar requirements for the applied membranes. The chemical, food and bio- and oil industries require energetically demanding separation processes for production and for wastewater treatment, preferably for reuse [16]. Switching these to environmentally friendly processes, such as NF, enables the reduction of energy costs and CO_2_ emissions. The increasingly growing body of nanofiltration research and the growing membrane market share (see Figure 1) suggests that the technology is popular and holds promise of further development to support green and sustainable separation processes [4,17].

### 1.2. Nanofiltration for Wastewater Treatment Applications

For tertiary municipal wastewater treatment by nanofiltration, the adequate retention of organic micropollutants is crucial (2–300 Da MWCO), coupled with a high salt passage, to prevent salt concentration in the bioreactor or other micropollutant-removing processes, to where the concentrate is usually returned. In addition, to curb energy requirements, high permeability is also an important requirement [19,20,21]. Therefore, there has been a significant effort made by the academic research community to develop highly permeable membranes with high MP rejection values and to further improve MP removal and salt/MP selectivity of NF membranes [22,23].

Industrial wastewaters vary greatly in terms of their harmful and harmless inorganic and organic composition, and can have extreme pH values, necessitating the use of specialty membranes [24,25]. Furthermore, it is advantageous if the properties of the membranes (selectivity, zeta-potential, MWCO) can be fine-tuned for the separation process.

In wastewater treatment, fouling is a crucial issue which can limit the applicability of a membrane process [3,26]. Zhao et al. pointed out that pretreatment is crucial for conventional NF systems to achieve reasonable fouling rates; on the other hand, improper pretreatment (such as too-high residual aluminium content from coagulation–flocculation, antiscalant overdose, etc.) can lead to a higher fouling rate than no pretreatment [27]. In terms of membrane materials, polymeric TFC NF has been widely adopted in wastewater treatment; however, its propensity to foul and long-term stability remain major concerns [28,29].

In 2008, Van der Bruggen et al. identified six challenges for NF which are still applicable today [30]. Of the six, four are directly related to the membranes itself: (1) membrane fouling and remediation, (2) improving selectivity, (3) membrane lifetime and chemical resistance (broad pH range, resistance to oxidation agents—e.g., hypochlorite) and (4) insufficient rejection for specific compounds (e.g., micropollutants). Additionally, the treatment of concentrates (5) is an intrinsic problem of all pressure-driven membrane processes, but can be somewhat improved by membrane development in the case of nanofiltration; recovery can be moderately enhanced by improving the selectivity (e.g., high salt passage, high micropollutant rejection), therefore limiting the osmotic potential of the concentrate stream. The treatment of concentrates originating from wastewaters present a challenging technological problem, involving costly and sophisticated technologies such as electrode ionization or advanced oxidation processes [31].

In 2017, Freeman et al. called for more selective membranes instead of primarily focusing on increased permeance, preferably via environmentally friendly production processes, such as solvent-free, aqueous-based fabrication [8]. In 2020, Zhang et al. highlighted the research effort focused on enhancing the selectivity of thin-film composite (TFC) NF membranes [32]. Our review focuses on polyelectrolyte multilayer NF membranes, which exhibit considerable improvement over conventional TFC NF membranes in the aforementioned areas, making them ideal candidates for municipal and industrial wastewater treatment processes.

### 1.3. Polyelectrolyte Multilayer Membranes: The Layer-by-Layer (LbL) Method

**Figure 2:**
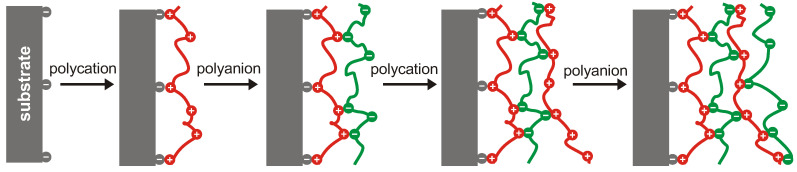
The layer-by-layer coating process [33].

Decher first reported the layer-by-layer assembly of polyelectrolyte multilayer (PEM) nanofilms by adsorption from a solution in 1997 [33]. As seen on Figure 2, the LbL process involves alternatingly coating a surface with polycations and polyanions to build up a multilayer. The thickness of the film is determined by the number of coating steps, and if substrate charge densities are small, each coating step increases the surface charge density and therefore the amount of oppositely charged polyelectrolytes in the next step, until the linear deposition regime is reached, where the layer thickness becomes independent of the substrate. Besides the number of layers, layer thickness can be effectively controlled by the concentration of NaCl (ionic strength) in the polyelectrolyte solutions used for layering [34].

Krasemann and Tieke first highlighted the separation possibility via a LbL PAH/PSS PEM membrane, and explained the mono/divalent separation by the Donnan effect [35]. Shortly thereafter, Harris et al. published similar results [36]. The application of an NF membrane was first mentioned in 2003 by Stanton et al. [37]. PEMs, applied on an appropriate membrane support surface, can act as active separation layers, making the resulting membranes tighter. LbL modification has been applied successfully on MF support to enable the 3-log removal of viruses [38], to notably enhance the ion selectivity of electrodialysis membranes [39,40,41,42], to create forward osmosis [43], pervaporation [44,45] and RO membranes (with limited practical applicability) [46], and furthermore to improve fouling resistance of NF [47,48] and RO [49,50,51] membranes.

**Figure 3:**
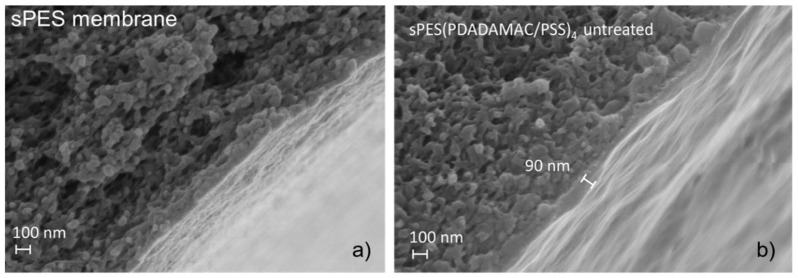
SEM images of sPES membranes; (**a**) uncoated membrane, (**b**) LbL-coated sPES(PDADMAC/PSS)4 membrane (figure taken from [52]).

The most common LbL membrane modification in the literature is preparing a nanofiltration membrane on an ultrafiltration support [4,5,45,53,54,55,56,57]. As seen in Figure 3, the multilayers form a ~100 nm thick homogenous film. PEM NF membranes generally have a significant organic rejection coupled with a high salt passage, which is ideal for micropollutant-removal [21]. Furthermore, a high selectivity between organic components (e.g., a notably high maltose/glucose selectivity of 46 [58], glucose/raffinose selectivity of 100 [59]) can be utilized for other fields where a high selectivity is important, such as food industry applications. Studies show that a spontaneous self-healing mechanism can repair physical damage on polyelectrolyte complex structures [60,61]. In principle, PEM NF membranes also exhibit this phenomenon [56], but they have not been extensively studied. This phenomenon is interesting because it can lead to high membrane lifetimes.

The intensive research of the past 20 years in this field has not only produced very interesting findings in the laboratory, but in the past few years, novel products based on hollow fiber (HF) PEM have also appeared on the market (Pentair HFW 1000 in 2013 [62], NX Filtration dNF40 and dNF80 in 2017). This underlines the importance of the research in this field.

## 2. Parameters of the Layer-by-Layer Fabrication Process

The versatility of the LbL method enables the setting of various parameters during the preparation process and various post-treatment techniques to fine-tune the properties of the end product. This membrane development process is important for achieving high micropollutant/salt selectivity coupled with fouling resistance and a long membrane lifetime.

### 2.1. Substrate

LbL coating an ordinary UF membrane, e.g., polyethersulfone (PES), yields an NF membrane with notable rejection properties, but the PEM layer is not particularly stable for longer timeframes and not resistant to backwashing [63]. However, sulfonated PES UF membranes, which have a charged surface, can withstand multiple, high-pressure backwashing cycles without any decline in membrane performance [63,64].

UF membranes more commonly have a hollow fiber structure, which lends itself to the practicality of producing HF NF membranes via the LbL method. The most common substrate is a tight hollow fiber ultrafiltration membrane prepared from sulfonated PES (which is the basis of the available commercial PEM NF membranes as well), which lends it a stable negative surface charge. Hydrophilic PES can also be used [65], but with limited pH and backwash stability [63]. Commercial nanofiltration membranes can also be modified by adding a top layer of PEM to enhance properties such as selectivity and fouling resistance [10].

Solvent-resistant NF membranes can be prepared, which have long-term stability in aggressive solvents such as CAN, DMF and THF by using a hydrolyzed polyacrylonitrile as a substrate to deposit PAH/PAA multilayers [66]. The utilization of a tubular ceramic support can also lead to solvent-stable membranes; furthermore, the deposition of a PAH/PSS multilayer leads to a membrane with lower MWCO than expected, yielding a ceramic RO membrane [67]. Transition metal rejections of ~100%, and >98.5% fluoride and >94% nitrate rejections, were achieved at optimal pressures [68]. Ceramic membrane support with PDADMAC,/PSS. Radeva et al. also successfully produced PEM membranes on ceramic alumina UF supports, obtaining NF membranes with 50–95% rejections for various micropollutants [69,70].

### 2.2. Polyelectrolyte Properties

PAH/PSS membranes exhibit a high mono/divalent selectivity for cations, much more than PDADMAC/PSS films, which have a higher degree of swelling, and therefore a higher water flux [71]. PAH/PAA multilayers have lower fluxes coupled with a high rejection of small molecules, but because of their imperfections, the pore-size distribution is not ‘sharp’; therefore, the MWCO is quite high [72,73]. If the molecular weight of the polyelectrolytes used is lower than the MWCO of the membrane, there is no initial rise in rejection as the pores are still too broad. When polyelectrolytes begin to accumulate on the membrane surface, forming an even layer, the rejection of MgSO4 increases [74].

De Grooth et al. revealed that adding a polyzwitterion layer between the polycation and polyanion moderately enhanced the dielectric exclusion of micropollutants and increased the salt-dependent permeability of the membrane [75,76].

Chiral separations can be achieved by utilizing optically active polyelectrolytes to build the PEM membrane, as confirmed by the separation of L and D ascorbic acid [77].

Organic polyelectrolytes can be substituted by inorganic species such as graphene oxide, which combined with PDADMAC exhibited stability in extremely acidic and saline conditions [78,79].

### 2.3. Layer-by-Layer Deposition Parameters

The number of polyelectrolyte layers is unequivocally a crucial parameter which can control the layer coverage (full or partial PEM coverage of the substrate) and thickness of the multilayer, which affect the flux and selectivity properties of the resulting membrane. LbL can be a quite lengthy process, with layer numbers reaching as high as 100, but 2–3 bilayers can already result in a membrane which is in the nanofiltration range [80]. The novel single-step method (by precipitating the polyelectrolyte complex during the spinning process) of Gherasim et al. produced NF membranes which had properties comparable to top-of-the-line LbL membranes: 7.6 Lm^−2^ h^−1^ bar^−1^ pure water flux and over 90% rejection for both divalent anions and cations and MWCO of ~300 Da [81]. Although overall the LbL method is well scalable, environmentally friendly and cost-efficient [82], lowering the number of bilayers can further improve fabrication costs and is therefore an important research goal.

Most NF membranes have a negative surface charge, but for softening purposes, a positive-surface-charged membrane can take advantage of the Donnan effect [56]. Many PEM NF membranes can achieve a hardness rejection above 95% while having a high NaCl passage [83]. The strong local electric field caused by polyelectrolyte bilayers in the PEM explains the higher selectivity between mono- and divalent ions compared to conventional membranes [84]. The top layer of the PEM can be either a polycation or polyanions, consequently determining the surface charge, which affects the basic Donnan exclusion properties of the membrane [71]. The alternating surface charge of the membrane created by adding an extra layer of the oppositely charged polyelectrolyte leads to large differences in permeability, zeta potential and divalent ion rejection; this is called the odd–even effect [65,71].

The salt concentration of the polyelectrolyte solutions used for the LbL process has a huge effect on the thickness and the density (and thus on the water permeability) of the resulting multilayer [85]. The type of salt anion (Cl^−^, Br^−^ or SO_4_^2−^) used in the LbL process also has a small but significant effect on the PEM membrane properties [51,86]. Kamp et al. combined low ionic strength in the polycation and high ionic strength in the polyanion coating solutions, which resulted in a PEM NF membrane with excess negative charges, which produced Na_2_SO_4_ rejections of above 99%, and a lower rejection of NaCl than for MgCl_2_ [87]. PEMs prepared at lower ionic strength exhibited decreased hydration, resulting in a greater capacity for rejecting micropollutants than PEMs prepared at higher ionic strength [21].

Burke and Barrett studied the influence of the layer number, the pH and salt concentration at assembly on the stability on PAH/PAA multilayers. PAA became a stronger acid and PAH a stronger base by 1–4 pK units in the multilayer assemblies. The acid and base strength in the films both increased by increasing the number of layers [88]. This phenomenon explains the much wider pH stability of PEM membranes than what one would expect from the pK_a_ values of the polyanions and the pK_b_ values of polycations. Multiple studies showed that the pH at the deposition step had a significant effect on the separation properties of the resulting membranes [54,89,90].

### 2.4. Post-Treatment after LbL

After building the PEM, annealing procedures can help stabilize and tune the properties of the resulting membranes by keeping the membranes in a specified salt concentration or pH at a certain temperature. The surface roughness of PEM membranes can be reduced by salt annealing [91,92,93,94], which leads to better nanofiltration membrane properties. Abtahi et al. showed that annealing PAH/PAA PEMs in 0.1 M NaNO_3_ significantly increased the micropollutant rejection of the membranes without compromising the permeate flux [95]. Salt annealing increases polyanion PSS content of the outer layer in the case of PDADMAC/PSS membranes, which leads to a more negative surface charge, thereby decreasing contact angle and increasing pure water flux and sulfate rejection [96,97]. Ng et al. affected the PDADMAC/PSS PEM stability against backwashing by annealing via heat treatment [98]. Acid doping can also be used to tune the hydrophilicity and membrane conductivity, as was shown on LbL modified polyaniline membranes [99].

The covalent crosslinking of polyelectrolytes in the multilayer is another important post-treatment option, which generally leads to more stable and tighter membranes, typically accompanied by a reduced flux [100,101]. Saeki et al. demonstrated that cross-linking solved the problem of the destabilization of the PEM layers at higher salt concentrations [102]. In this study, it is notable that amine coupling somewhat counter-intuitively increased the water permeability while slightly decreasing the MgSO4 rejection. PEM membranes are quite sensitive to both cationic and anionic surfactants, which can be problematic in the case of treating produced water [103,104]. Crosslinking PAH/PSS membranes can yield a stable structure which retains more than 80% of its mass [104]; furthermore, their long-term acid resistance also increased with glutaraldehyde crosslinking as well [105]. Crosslinking can also enable the LbL fabrication of a membrane which contains only a single polyelectrolyte, e.g., an anionic carboxymethyl cellulose with PVA, crosslinked by glutaraldehyde [106].

The ion-imprinting technique can also enhance selectivity and permeability, as shown by Chen et al.: intercalating certain ions during the LbL process, crosslinking and then washing them out [107].

### 2.5. Adding a Function to the PEM

PAH/PAA with carbon nanotubes exhibited a stronger chlorine resistance compared to conventional RO membranes [108] and PDADMAC/PSS combined with carbon nanotubes led to improved fouling resistance compared to UF; after protein fouling, flux can be restored by water flush [109].

The AquaporinZ protein can also be incorporated into a PEM active layer: in this manner Sun et al. managed to reach an average two-fold increase of the water permeability on PAH/PSS LbL membranes [110].

Dizge et al. prepared a biocatalytic NF membrane by creating a PDADMAC/PSS PEM by the LbL method and immobilizing trypsin on the membrane surface by electrostatic attraction or covalent bonding. The enzymes mitigated protein fouling, which is a useful feature in the food industry, among other uses [111]. Varga et al. successfully demonstrated micropollutant rejection via laccase immobilization on membranes [112]. Zdarta et al. achieved over 70% estrogen removal via laccase immobilized in PEM [113].

### 2.6. Asymmetric PEM Membranes

In 2019, a novel approach to creating PEM membranes emerged: the coating of the loose PEM by an extra outer, ultrathin layer, which acts as the active filtration layer, with the loose multilayer acting as the support [72]. This has led to outstanding membrane properties: breakthroughs in the permeability/selectivity and organic/salt selectivity tradeoff limits.

Applying a rapid co-deposition of polydopamine/polyethylenimine (PDA/PEI) on a PEM assembled on a PES support, optionally crosslinked with glutaraldehyde, yields a chemically stable membrane with excellent performance characteristics during testing with municipal wastewater used directly as feed [100]. One problematic factor emerged in this study: most membranes exhibited decreased performance after being subjected to a moderately alkaline (pH = 10) cleaning. The PDA/PEI-terminated and cross-linked membrane proved to be the most selective while maintaining a high flux. It also proved to be the most stable during alkaline cleaning, and did not exhibit any significant performance decline, but unfortunately the study only employed three cleaning cycles [100].

Ouyang et al. showed in 2008 that the rejection/flux compromise can be overcome by capping PDADMAC/PSS films with a bilayer of PAH/PSS to achieve a high flux in combination with a high recovery [20]. This promising route was not explored further until 2019, when Brinke et al. achieved the lowest top active layer for membranes ever reported in the literature [72]. This asymmetric structure, a PAH/PSS ultrathin PEM on a loose PAH/PAA PEM, showed remarkable selectivity and micropollutant rejection (98%) while retaining a high flux rate (12.8 Lm^−2^ h^−1^ bar^−1^).

Nafion-terminated PAH/PSS membranes have achieved neutral micropollutant (SMX, bisphenol A) rejections which surpass common RO membranes. The hydrophilicity of these membranes is relatively low (consequently, the swelling is also minimal); therefore, the water flux is lower comparable to RO membranes, but the sodium salt rejection is significantly lower (only minimally larger than for the non-modified LbL membrane), which has advantages for many applications [114].

## 3. Advantages of PEM Membranes

### 3.1. Superior Selectivity

The monovalent/divalent ion and micropollutant/salt selectivity of PEM membranes are often higher than TFC membranes [21,72], which can be further tuned by optimizing the LbL process parameters (no. of layers, salt concentration) to achieve precise salt rejection values with unprecedented precision [115,116,117].

A high halide selectivity was achieved by Su et al. by applying the PEM on alumina support. The high selectivity did not compromise the flux, which was three-fold higher compared to commercial NF membranes [118]. Our results published in 2020 also point to a high selectivity: PEM NF provided the highest selectivity between acetate, propionate and butyrate during the filtration of an artificial anaerobic effluent wastewater, out of four compared separation technologies (NF, RO, FO and supported liquid ionic membrane) [119], far surpassing the selectivity of TFC NF membranes [120]. PSS/PAH NF membranes were capable of achieving exceptional Mg^2+^/Li^+^ separation, irrespective of the solution conditions and whether crosslinking was applied or not, outperforming commercial NF membranes [121]. The selectivity of a PDADMAC-terminated PDADMAC/PSS LbL-modified Synder NFG membrane demonstrated quite high (over 30) Mg^2+/^Na^+^ selectivity [48]. The F/Cl selectivity of the NF270 membrane was increased from 1 to 2.7 by adding 8 PDADMAC/PSS bilayers [47]. Wang et al. measured similar organic pollutant rejection for the widely used NF270 membrane and (PDADMAC/PSS)6 and (PDADMAC/PSS)4 PEM membranes, measuring a much lower (~20%) rejection of NaCl and CaCl_2_ in the case of the latter [122].

Compared to the Filmtec NF90 membranes, Pontie et al. achieved superior lithium–calcium selectivity with the commercial PEM dNF40 membrane, while having an order of magnitude lower energy consumption [116]. (PAH/PSS)4 PEM membranes with dopamine intercalation exhibited additional improvement in selectivity (Li^+^/Mg^2+^ selectivity from 15.6 to 37.8 and Na^+^/Mg^2+^selectivity from 11.0 to 19.1) while enhancing water permeability (13.5 to 21.9 LMH/bar) [123]. Qin et al. investigated the recovery of CuSO_4_, ZnSO_4_, NiCl_2_ and CdCl_2_ from an artificial wastewater model solution by using a PEI/PSS PEM membrane, achieving metal ion rejections between 95–98%, with permeation fluxes ranging between 19 and 24 LMH [124].

Naturally occurring polyelectrolytes have also been successfully applied as building blocks for highly selective PEM membranes and demonstrated excellent dye/salt separation, such as lignosulfonate/dopamine [125] and carboxymethyl cellulose/polyethylenimine, achieving 99.4–99.8% dye rejection values [126]. The latter had a strongly positive surface charge, which led to the following salt rejection order: MgCl_2_ > CaCl_2_ > KCl > NaCl > MgSO_4_ > Na_2_SO_4_ at neutral pH. Chitosan/PAA membranes can also achieve tailored salt selectivity; however, even after thermal annealing, severe degradation occurred within 5 operation days, which was largely alleviated by crosslinking [127].

**Figure 4:**
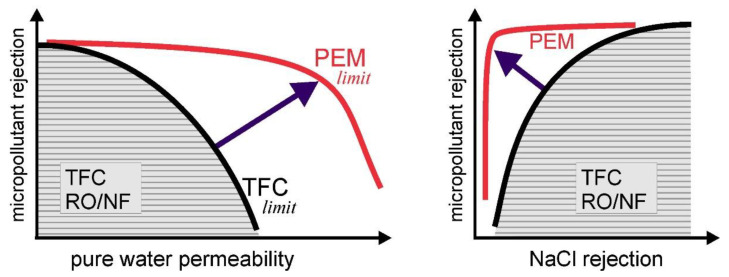
Schematic, qualitative representation of micropollutant//NaCl selectivity of PEM NF membranes compared to common NF and RO membranes (scheme based on data from ref. [72,100,114], micropollutants in the 200–400 Da range).

The ultrathin separation layer of asymmetric PEM membranes [72] opens up a new dimension for enhancing selectivity, further extending the MP/water and MP/salt selectivity advantage of PEM membranes, as shown in Figure 4.

Table 1 summarizes the selectivity advantages PEM membranes have over polyamide NF membranes.

### 3.2. Fouling Resistance

Fouling is a major issue for membrane separation processes, thus making the development of low-fouling NF membranes a priority, particularly with regard to wastewater treatment [50,128]. It is commonly anticipated that fouling increases with the decrease in hydrophilicity of the membrane surface [29], although increasing the surface charge also plays an important role, and often the two are achieved with the same modification [129,130].

Polyelectrolyte surface modifications can help reduce fouling by both of the aforementioned effects [131]. However, Reiss et al. found that electrostatic repulsion increased fouling correlated with a more negatively charged membrane surface [132], therefore the relationship between hydrophilicity and fouling resistance remains a somewhat ambiguous topic. Teow et al. reviewed current antifouling strategies applied to TFC NF membranes for wastewater treatment, concluding the importance of high hydrophilicity, surface roughness and charge [133]. The surface chemistry of PEM membranes offer a solution to all these problems [134,135]. The permeability of PEM NF membranes is on par with state-of-the-art TFC membranes (e.g., NF270), yet they appear to provide an edge when processing high-fouling feedwaters. Furthermore, the hollow fiber geometry offers added advantages: the absence of colloidal fouling, the possibility of cleaning by backwashing and air sparging [135,136].

The fouling resistance is even greater compared to UF because there is a smaller chance of pore blocking with the homogenous thin layer of polyelectrolytes on the inner surface of the capillaries. The flux decline was much steeper in the case of an UF membrane, compared to NF when fed with the same water source [137].

Scaling is significantly less problematic for PEM NF membranes compared to polyamide NF because of the low salt rejection. Compared to NF270, PDADMAC/PSS PEM exhibited many times less gypsum scaling [122].

PEM membranes possess intrinsically antimicrobial properties [138], and the biofilm resistance can be enhanced by the addition of additional antimicrobial molecules, such as norspermidine [139], copper ions or silver nanoparticles [140].

In 2019, Virga et al. developed a stable HF NF membrane for produced water treatment. By cross-linking their (PAH)/PSS multilayers with glutaraldehyde, surfactant-stable membranes were achieved. The membrane showed excellent oil removal (>98%) for two synthetic produced waters and TOC retention of 96.5% and 83% for a cationic-containing surfactant and an anionic-containing surfactant, respectively. Flux recovery after cleaning with pure water was fully achieved for the cationic solution (100%) and it was possible to recover 80% of the initial flux while using the anionic surfactant solution [104].

Virga et al.’s results demonstrate that fouling of PEM-based NF membranes during produced water treatment is mainly due to membrane active layer fouling caused by surfactant uptake inside of the PEM coating, rather than due to cake layer formation. Indeed, it is not the surface chemistry of the membrane that determines the extent of fouling, but the surfactant interaction with the bulk of the PEM. A denser multilayer which would stop these molecules would benefit produced water treatment [141].

Table 2 demonstrates that PEM membranes exhibit superior fouling resistance for all types of fouling, apart from their lower tolerance to ionic surfactants.

### 3.3. Chemical Stability and Cleaning

The chemical resistance of PEM membranes is ultimately determined by the supporting layer and the chemical properties of the polyelectrolytes. This enables the extension of pH tolerance and chlorine tolerance.

Remmen et al. demonstrated that PDADMAC/PSS membranes prepared on a PES UF substrate were stable at pH 1.5 and had comparable Sc rejection to commercial acid-resistant NF membranes while exhibiting a much higher flux. Decreasing the pH down to 0.1 had a detrimental effect on the membrane [148]. Aluminum recovery with over 95% rejection was also demonstrated with PDADMAC/PSS membranes with a sufficiently low phosphoric acid retention (below 10%), achieving a higher flux compared to commercial acid-resistant membranes [149]. Achieving an even more strongly acid-resistant PEM membrane is desirable, since the flux of acid-resistant LbL membranes tested for phosphorous recovery is notably higher than comparable commercial acid-resistant TFC membranes [149,150]. In a recent paper, Elshof et al. demonstrated the long-term stability of PDADMAC/PSS membranes prepared on a sulfonated PES UF substrate in both extreme acidic and alkaline solutions (pH = 0 and 14) [151], which suggests that these membranes are ideal candidates for acidic and basic CIP solution recovery applications. This is in stark contrast with the findings of Remmen et al., who found that sulfonated PES-based PDADMAC/PSS membranes were less stable than PES-based ones when exposed to 15% phosphoric acid [52].

Comparing strongly basic/acidic PEM (PDADMAC/PSS) and weakly basic/acidic PEM (PAH/PAA)-based membranes, Junker et al. noted that slight variations in structure and performance were observed for the former, but large variances were observed in the latter case when varying the pH of the feed solution [152]. It should be noted that even weakly acidic/basic polyelectrolyte pairs can form rather pH-stable multilayers, because the apparent pK_a_ of the polymers in the polyelectrolyte complex is much higher compared to the individual polymers, leading to pH tolerance values approximately 2 pH units wider for PEMs compared to the original pK_a_ values of the building block polymers [153,154]. The stability of PEMs prepared from the weakly basic PAH and strongly acidic PSS can be further enhanced by crosslinking, leading to excellent stability in concentrated phosphoric acid (pH = 0.7) [105].

De Grooth et al. demonstrated that PDADMAC/PSS multilayers exhibit excellent long-term chlorine tolerance, and the resulting PEM’s chlorine resistance is in the same range as the substrate UF membrane (250,000 ppm hours at pH 11). On the other hand, the other most commonly used PEM, PAH/PSS, exhibited five times lower chlorine tolerance [63], which is still much higher than common polyamide NF and RO membranes. Nevertheless, the long-term stability of the membranes are served better by minimizing exposure to oxidative agents in the case of the most common supporting agents, PES and sulfonated PES, which also have a limited chlorine tolerance [155]. Ilyas et al. tested PAH/PAA PEM membranes prepared on PAN support in aggressive solvents such as THF, DMF and CAN, demonstrating stable operation over 50 h [66].

Chemical cleanability is crucial for maintaining a high permeability and rejection for NF membranes, especially when treating high-fouling-potential feedwaters [156]. Caustic cleaning can have a profound effect on the properties of TFC NF membranes; Simon et al. found a pore size increase for an NF270 membrane after caustic exposure [157]. However, when treating fouled membranes fouled by real feedwater, Huang et al. found that although the fleeting pore size expansion could be observed after NaOH exposure, repeated cycles of caustic cleaning led to irreversible fouling of polyamide membranes, which could be mitigated by the addition of dodecyl sulfate (SDS) [158]. This underscores the importance of optimal cleaning procedures for achieving optimal membrane performance.

The range of cleaning procedures available for HF PEM NF membranes exceeds that which is usable for TFC membranes. Backwashing is available with 6 bar transmembrane pressure (TMP), while forward flushing is available up to 2 m/s, and these two procedures can be combined. Because of the larger pH range and chlorine tolerance, aggressive chemical cleaning protocols can be implemented. However, it is important to note that ionic surfactants can damage PEMs without crosslinking (see Section 4.2). All long-term pilot studies to date (detailed in Section 5) applied a relatively frequent CIP procedure to avoid irreversible fouling; therefore, no information is available in the literature regarding whether fouling converges to a steady-state secondary cake layer which stabilizes flux at a lower level, or whether any irreversible fouling occurs at all.

Table 3 summarizes the resistance of PEM membranes to various chemical stressors, clearly illustrating their advantages over polyamide NF membranes.

## 4. Disadvantages of PEM Membranes

Although PEM-based membranes are known for their chemical robustness, they still have certain weaknesses that can limit their applicability to certain wastewater treatment applications.

### 4.1. Limitations with High-Salinity Feeds

High ionic strength can damage membranes, but the exact limits were not mapped yet. A highly concentrated salt solution negates the entropic gain resulting from the PEM structure and can dissolve the layers. Ilyas et al. demonstrated that backwashing a PAH/PAA membrane with pH 3, 3M NaNO3 resulted in almost all recovery of the membrane resistance nearly equal to that of the pristine membrane [162].

### 4.2. Sensitivity to High-Affinity Ionic Species

Surfactants are important cleaning agent for removing organic fouling in the case of conventional TFC membranes [158,163,164]. However, ionic surfactants are capable of destroying PEMs by forming complexes and dissolving the polyelectrolyte molecules [165], which is a limiting factor for treating produced water [166]. In the case of a ceramic substrate, the PDADMAC/PSS multilayer was also damaged by SDS, but interestingly, a pH above 10 also made the PEM more permeable to micropollutants [70].

The exact limits of the effect of ionic surfactants having low concentration (below critical micellar concentration) have not been determined to date. Recent developments suggest that PEM membranes will be able to tackle high-salinity brine feedwaters and surfactants; a cross-linked LbL membrane prepared by Virga et al. showed notable stability various ionic surfactant solutions which disintegrates most PEMs [104].

A few studies suggest that another advantage of PEMs is the ability to remove irreversible fouling via dissolving the active layer by a chemical trigger (surfactants, backwash with a high ionic strength solution) [49,80,162]. However, this solution is difficult to put into practice [82] because of two reasons: this would lead to a long and complicated CIP procedure and commercial membrane producers tend to keep the details of their membrane preparation procedures confidential.

Transition metals, due to their strong affinity for polyanionic species, can cause problems by binding to them through chelation. Copper, in particular, can be problematic in the case of polyimide membranes, as it disrupts the self-healing behavior of the multilayer [60]. Jährig et al. experienced substantial iron and manganese fouling during long-term pilot testing of the experimental Pentair HF-TNF membrane when directly feeding surface water, which decreased substantially by switching the feed to anoxic bank-filtered river water [167]. The membrane could be restored by a reducing acidic CIP, for which ascorbic acid was added to the HCl solution, but oxalic acid is also an effective, but cheaper alternative [167].

### 4.3. Pressure Limitations

The 6 bar pressure limit for commercial membrane modules (Pentair HFW1000 and NX Filtration dNF) is not an issue when treating low-ionic-strength feedwaters due to the high permeability, low fouling and the high passage of inorganic salts, which eliminates the need for high trans-membrane pressures in most applications. However, this may pose a practical limitation for applications involving high-osmotic-pressure feedwaters, such as high-strength industrial wastewaters and for high-recovery filtration of municipal wastewaters.

### 4.4. Concentration Polarization

A downside of hollow fiber NF is the propensity for concentration polarization, which lowers the apparent rejection of a membrane module and lowers the flux [135]. This can be overcome by high crossflow over ~0.5 m/s; however, it comes at a significant energy demand [168], which is comparable to the energy need of the pressurization of the feed to TMP. For hollow fiber membranes, turbulence is achievable only for short (15–30 cm) lab scale modules with a very high pressure loss over the module (over 1 bar) [135].

## 5. Studies of PEM NF Involving Real Wastewater

The laboratory studies discussed in the preceding chapters demonstrate the promising nature of PEM NF membranes for wastewater treatment. Though the number of studies involving real wastewater feeds is limited at present, the results are promising. Jonkers et al. stressed the importance of using realistic feed streams over the artificial compositions used commonly in academic studies [135]. This is supported by the authors’ recent study involving NX Filtration dNF40 membranes for beer dealcoholization, which is not a wastewater application, but still poses similar challenges to the membrane separation process (high organic loading, relatively high osmotic pressure). After only a short (2 × 5 h) beer filtration duration and caustic cleaning, the Na_2_SO_4_ and MgSO_4_ rejection of the PEM membranes markedly improved with only insignificant permeance loss [169].

The efficacy of phosphoric acid recovery from leached sewage sludge ash was investigated by Paltrinieri et al., who concluded that LbL-modified membranes improved permeability and recovery of phosphoric acid in comparison to a commercially available, acid-resistant nanofiltration (NF) membrane [150]. Sanyal et al. conducted a short-term laboratory-scale study which exhibited the utility of coating a commercial NF270 membrane for the treatment of real wastewater treated by electrocoagulation, achieving similar COD reductions as an NF90 or BW30 membrane yet displaying significantly lower fouling [170]. In the layering of clay nanoplatelets onto a commercial PES membrane, the PEM film produced hybrid nanostructured membranes with high fouling resistance and higher COD reduction compared to the non-modified PAH/PAA PEM membrane, and both outperformed the commercial NF270 tested using real wastewater treated by electrocoagulation [171].

Several case studies are available from NX Filtration BV: dNF40 membranes were used for 3 years to treat a challenging industrial wastewater for RO feed pretreatment [172], a pilot unit has been operating in Twente for tertiary treatment of municipal wastewater [173] and a full scale plant was started in 2022, employing dNF membranes to produce drinking water from municipal wastewater effluent [174]. Through a series of long-term tests carried out with surface water and on biologically treated effluent from a municipal wastewater treatment plant, NX Filtration’s dNF membranes provided an average retention of 95% for PFAS20 (EU regulated) and PFAS4 (Swedish regulatory) group substances [175]. In a brewery, dNF40 membranes were used for treatment of RO concentrate and during the 2-month operation period, no observable fouling was recorded [135].

Wagner et al. investigated the option of using a dNF40 membrane to filter discharged cooling tower water. They observed a 95% TOC reduction for greywater and 97% reduction for wetland-pretreated feed, coupled with a negative rejection for chloride and nitrate [176]. Benzotriazole removal was over 97% in all tried process parameters (TMP 1–3 bar, crossflow: 0.5–1 m/s) for experiments conducted in short time scales; however, the longer (35–48 h) experiments showed a breakthrough of benzotriazole and practically no rejection. This result underlines the need for prolonged experiments with adequate time. An important question remains unanswered in this study: how the hollow fiber NF pretreatment affects the stable functioning of RO membranes in the long run. The high TOC removal observed might lead to significantly less organic- and biofouling [177], and the small pore size to negligible colloidal fouling. A long-term pilot could provide more information about these questions.

Pilot studies involving PEM membranes for the direct nanofiltration treatment of challenging feedwaters of high-COD surface waters for potable water applications also provide insight into how PEM NF membranes could perform in the tertiary treatment of biologically treated wastewater. The pilot study conducted by Keucken et al. conducted with a full-scale Pentair HFW1000 module on a 9 ppm DOC lake water demonstrated the stability of the PEM membrane; after a year of continuous use, no irreversible fouling was noted and the membrane autopsy detected no substantial changes compared to virgin samples besides a slight contact angle increase [14]. Keucken et al. also tested the HFW1000 feed and bleed pilot system to optimize filtration parameters [168]. The permeate quality (monitored by organics removal rate) increased up to 0.75 m/s crossflow but decreased around two-fold when the recovery rate was increased from 50% to 80%. Interestingly the fouling rate was affected just by filtration flux. During a 9-month pilot study, Köhler et al. compared the loose HFW1000 PEM NF membrane to ultrafiltration combined with granular-activated carbon treatment on surface water filtration [15]. Nanofiltration yielded superior and more stable TOC and micropollutant removal during the study period, to which similar conclusions were drawn by the pilot study of Lidén and Persson [178]. A long-term pilot study comparing coagulation combined with ultrafiltration and direct PEM nanofiltration to the conventional physiochemical treatment conducted by Lidén et al. found that the NF yielded the lowest greenhouse gas emissions while providing the highest product water quality [137,179]. Jährig et al. observed iron scaling during the treatment of anoxic bank-filtered water [167]. This phenomenon could be problematic for high-transition-metal-containing wastewaters; how other PEM membranes composed of different polyelectrolytes would handle this problem is an interesting research question.

## 6. Conclusions

Wastewater treatment is a demanding task for NF membranes, requiring high selectivity, fouling resistance and chemical stability. Numerous laboratory investigations presented in this review suggest that PEM NF membranes outperform state-of-the-art polyamide and polyimide TFC NF membranes in these areas, as shown in Section 3. Additionally, the layer-by-layer process provides flexibility to further improve membrane properties, as detailed in Section 2. The limitations of PEM NF membranes discussed in Section 4 highlights their limits for the treatment of industrial wastewaters with special compositions (very high salinity, surfactant content and high osmotic pressure) and the process limits regarding micropollutant separation from municipal wastewater, which places limitations to ideally high recoveries.

Selectivities of virgin membranes are well mapped out through studies involving fresh, virgin membranes tested in short-term laboratory experiments. However, questions remain regarding how membrane properties change as the PEM accumulates organic and inorganic ions with a high affinity to the PEM, and how permeability, selectivity and rejection are affected.

Fouling and membrane lifetime laboratory experiments employing synthetic water with added foulants also show promising results. Additionally, drinking water pilot studies conducted with challenging feedwaters have likewise demonstrated promising results.

PEM NF membranes have demonstrated excellent stability towards various harsh chemicals, such as acids (pH = 0), bases (pH = 14), chlorine and organic foulants. Numerous cleaning possibilities are available: backwash, combined with forward flush, with and without chemicals. Although long-term operation of PEM NF membranes with frequent CIP has been presented in studies, optimal cleaning procedures that balance effective cleaning with minimal consumption of water, energy and chemicals have still not been established.

Various NF performance indicators are employed even for synthetic test solutions, making it difficult to compare the performance of virgin membranes. Moreover, for industrial applications, decisions need to be made regarding performance with realistic feedwaters. While it is challenging to define and agree on such realistic reference feeds, future membrane development could also consider the effects that real feedwaters have on membrane performance to optimize membranes based on this information as well. The durability and longevity of PEM NF membranes is demonstrated by laboratory stress-tests (detailed in Section 3.3) and industrial case studies, as well as long-term pilot studies on challenging surface waters. However, detailed scientific studies about long-term performance involving municipal and industrial wastewater are lacking. Providing clear, tangible pilot performance results and application recommendations could significantly contribute to the widespread adoption of this emerging product of membrane science.

PEM-based membranes, besides their chemical robustness, also have some weaknesses, especially concerning swelling in high-salinity feedwaters and sensitivity to surfactants. The latter raises the question about the effect of natural organic matter and artificial surfactants in industrial wastewaters. Laboratory results point to chemical fouling having a significant effect on membrane performance indicators. Most lab and pilot studies were conducted at low recoveries, which is beneficial for studying the fundamental properties of these membranes. However, for wastewater treatment, both municipal and industrial, achieving a high recovery is essential for creating an economically and environmentally viable process, which can greatly affect the rejection values and fouling rate of the employed membranes. It is anticipated that data collection from pilot studies and full-scale plant operations will provide further insights into the most effective use of this technology for wastewater treatment.

## Figures and Tables

**Table 1:** Comparison of the rejection attributes of TFC and PEM NF membranes.

Rejection Attribute	Polyamide TFC	PEM
monovalent salt passage	medium	high
divalent salt passage (same charge as the membrane surface)	low	low/very low
divalent salt passage (opposite charge as the membrane surface)	low	very high
rejection of hydrophobic organics	low–medium	medium–high
organic/salt selectivity	low	high

**Table 2:** Comparison of the fouling properties of TFC and PEM NF membranes [122,133,142,143,144,145,146,147].

Fouling Type	Polyamide TFC	PEM
scaling	medium–high	low
organic fouling	medium	medium (but low tolerance to ionic surfactants)
biological fouling	high	low
colloidal fouling	high	very low

**Table 3:** Comparison of the chemical resistance of TFC and PEM NF membranes, with the most stable substrates considered for the latter [3,63,156,159,160,161].

Chemical Stressor	Polyamide TFC	PEM
pH	medium	medium for weak PE-s, high for strong PE-s
oxidants (peroxide, chlorine)	very low	high for certain PE-s
organic solvents	low	high
ionic surfactants	high	low

## Data Availability

No new data were created or analyzed in this study.
